# Inflammatory markers and bone health in postmenopausal women: a cross-sectional overview

**DOI:** 10.1186/s12979-019-0155-x

**Published:** 2019-07-10

**Authors:** Bolaji Lilian Ilesanmi-Oyelere, Linda Schollum, Barbara Kuhn-Sherlock, Michelle McConnell, Sonya Mros, Jane Coad, Nicole C. Roy, Marlena Cathorina Kruger

**Affiliations:** 1 Department of Nutritional Science, School of Food and Advanced Technology, College of Sciences, Massey University, Tennent Drive, Palmerston North, 4442 New Zealand; 2 Riddet Institute, Palmerston North, 4442 New Zealand; 3 Food Nutrition & Health Team, AgResearch Grasslands, Palmerston North, 4442 New Zealand; 4 Fonterra Research and Development Centre, Palmerston North, 4472 New Zealand; 5 BKS Consulting, Hamilton, New Zealand; 6 Department of Microbiology and Immunology, University of Otago, Dunedin, 9054 New Zealand; 7 High-Value Nutrition National Science Challenge, Auckland, New Zealand; 8 School of Health Sciences, College of Health, Massey University, Palmerston North, 4442 New Zealand

**Keywords:** Inflammatory markers, Cytokines, Chemokines, Ferritin, Osteo-immunology, Bone mineral density, Bone health, Postmenopausal women, Aging

## Abstract

**Background:**

Cytokines, chemokines, C-reactive proteins (CRP) and ferritin are known inflammatory markers. However, cytokines such as interleukin (IL-1β), (IL-6) and tumour necrosis factor (TNF-α) have been reported to interfere with both the bone resorption and bone formation processes. Similarly, immune cell cytokines are known to contribute to inflammation of the adipose tissue especially with obesity. IL-10 but not IL-33 has been linked to lower ferritin levels and anemia. In this study, we hypothesized that specific cytokine levels in the plasma of women with low bone mineral density (BMD) would be higher than those in the plasma of healthy women due to the actions of elevated levels of pro-inflammatory cytokines in inducing osteoclast formation and differentiation during senescence.

**Results:**

Levels of cytokines (IFNα2, IFN-γ, IL-12p70, IL-33) and monocyte chemoattractant protein-1 (MCP-1) were significantly higher in the plasma of the osteoporotic group compared to the osteopenic and/or healthy groups. Meanwhile CRP levels were significantly lower in women with osteoporosis (*P* = 0.040) than the osteopenic and healthy groups. Hip BMD values were significantly lower in women with high/detectable values of IL-1β (*P* = 0.020) and IL-6 (*P* = 0.030) compared to women where these were not detected. Similarly, women with high/detectable values of IL-1β had significantly lower spine BMD than those where IL-1β was not detected (*P* = 0.030). Participants’ CRP levels were significantly positively correlated with BMI, fat mass and fat percentage (*P* < 0.001). In addition, ferritin levels of women with high/detectable values of anti-osteoclastogenic IL-10 (*P* = 0.012) and IL-33 (*P* = 0.017) were significantly lower than those where these were not detected. There was no statistically significant association between TNF-α and BMD of the hip and lumbar spine.

**Conclusions:**

High levels of cytokines (IFNα2, IFN-γ, IL-12p70, IL-33) and MCP-1 in apparently healthy postmenopausal women are associated with bone health issues. In addition, an increase in levels of IL-10 and IL-33 may be associated with low ferritin levels in this age group.

**Trial registration:**

ANZCTR, ACTRN12617000802303. Registered May 31st, 2017, https://www.anzctr.org.au/Trial/Registration/TrialReview.aspx?id=373020

## Background

Cytokines are a large group of peptides and proteins, which are known to be involved in the signaling between the cells of the immune system [1, 2]. Examples of cytokines include interleukins (ILs), chemokines, interferons (IFNs), colony stimulating factors, tumour necrosis factors (TNFs), transforming growth factors (TGFs) and adipokines. Cytokines affect almost all biological processes in the body and can be either detrimental or beneficial depending on the amounts produced and conditions surrounding the production [1]. Cytokines play a critical role in the coordination of the immune system that is necessary for resolving bacterial and viral attacks on the immune system.

Furthermore, osteoporosis is a major public health concern which as a result of the demineralisation and weakening of bones leads to increased fracture risk [3]. Annually, reports suggest that osteoporosis causes more than 8.9 million fractures worldwide, resulting in an osteoporotic fracture every three seconds [4]. The burden of osteoporosis is therefore not limited to economic costs but also significant emotional and physical consequences, especially for middle aged and elderly men and women.

Aberrant or prolonged immune responses resulting in low-grade inflammation have been implicated in the pathogenesis of osteoporosis. In postmenopausal women, this is coupled with a decrease in oestrogen levels that leads to an increase in bone resorption [5]. Inflammation has also been related to indices of musculoskeletal health and several age-related diseases such as atherosclerosis, Alzheimer’s disease and cancer [6].

C-reactive protein (CRP) is known to be a sensitive systemic inflammatory marker. The production of CRP in the liver, which upregulates levels of cytokines such as IL-1, IL-6 and TNF-α has been observed to be positively correlated with bone resorption including hip and spinal bone loss in healthy pre- and postmenopausal women [7–9].

Cytokines are also known as crucial regulators of the adipose tissue metabolism especially in obese individuals with body mass index (BMI) and fat percentage above 25 kg/m^2^ and 32% respectively as an indicator of obesity. Cell types, pre-adipocytes and mature adipocytes are able to promote secretion of cytokines and chemokines associated with increased mRNA expression, notably in obese individuals [10, 11].

Infection, injury or trauma influences iron status. Ferritin is known as an acute phase reactant, a marker of inflammation. In addition, serum ferritin concentration is well-known as an important indicator of total body iron stores. The hormone, hepcidin is a major regulator of systemic iron homeostasis in the liver and it is induced during inflammation leading to leakage of ferritin into the plasma from damaged cells. This causes sequestration of iron and increased serum ferritin.

Cytokines in general are often complicated to research due to their synergistic effects and their ability to affect or enhance each other’s secretion. For example, IL-1 acts in synergy with TNF-α [12]. However, the cytokine network is significant in the regulation of the immune cells (primarily lymphocytes and macrophages) and the skeletal system where a natural balance is needed for bone metabolic homeostasis [13].

Based on observations and research in animal and in-vitro studies, cytokines have been classified according to their stimulatory or inhibitory effect on proliferation and differentiation of osteoclasts. Cytokines such as receptor activator of nuclear factor kappa-Β ligand (RANKL), macrophage colony-stimulating factor (M-CSF), IL-1, IL-6, IL-7, IL-11, IL-15, IL-17, IL-23, IL-34, monocyte chemoattractant protein-1 (MCP-1), TNF-α, TNF-β have been reported for their stimulating effects on osteoclastogenesis (OC) [6, 14–18]. Meanwhile, IL-1ra, IL-3, IL-4, IL-10, IL-12, IL-18, IL-27, IL-33, interferon IFN-α, IFN-β, IFN-λ, OPG and transforming growth factor-β (TGF-β) have been reported to have inhibitory effect on the proliferation and differentiation of osteoclasts [6, 15, 19–22]. These studies have indicated that low-grade inflammation, due to the effect of pro-inflammatory cytokines, impairs DNA repair and leads to cellular and immunological senescence as well as biological ageing. Increase of IL-31 has also been linked to decreased BMD in postmenopausal women [23]. However, the course of inflammageing is multi-factorial, resulting not only from immunosenescence but also from several factors such as dietary patterns, obesity and gut microbiota status [6].

The aim of this study was to measure levels of inflammatory markers in postmenopausal women. The outcomes of this study were; 1. The relationship between levels of inflammatory markers and lumbar spine, hip BMD, bone markers and osteoporosis status. 2. The relationship between inflammatory markers and obesity/adiposity. 3. The relationship between anti-inflammatory cytokines and ferritin levels in apparently healthy postmenopausal women. To our knowledge, no study has related these 15 immune markers to bone health in postmenopausal women.

## Methods

### Subjects

A total of 127 New Zealand European postmenopausal women aged between 54 and 81 years participated in the ‘BugsnBones’ study that took place in the Human Nutrition Research Unit at Massey University, Palmerston North campus from June to December 2017 [24]. Eighty-six women were then selected to participate in phase 2 for this study based on their bone strength for groups of healthy, osteopenic and osteoporotic women. Sample size was calculated using G*Power software version 3.0.10 and eighty-eight subjects were required for each group at a 90% power and an alpha of 5% for T-test. During this cross-sectional study, two subjects were excluded from the study, one due to consumption of a ketogenic diet and the other for health reasons. Subjects were recruited by advertisement on campus, the Wanganui Chronicle and by using a recruitment agency; Trial Facts (https://trialfacts.com/). The inclusion criteria were menopause of at least 5 years based on no menstruation. Exclusion criteria were presence of any systemic disease, food intolerances affecting the gastrointestinal tract, smoking and high intake of alcohol. Subjects with significant weight loss or weight gain within the past year were also excluded. All subjects were free living and apparently healthy. Written informed consent was obtained from subjects before commencing data collection.

### Anthropometric and body composition measurements of the subjects

The body weight of subjects was measured using the Detecto 437 eye-level weigh beam physician scale to the nearest 0.2 kg and standing height was measured using a stadiometer to the nearest 0.1 cm wearing light clothes and no shoes. Body mass Index (BMI) was calculated as weight divided by height squared (kg/m^2^) using the Quetelet’s index. Waist to hip ratio was determined by measuring the waist and hip circumference to the nearest 0.1 cm using a non-stretchable tape. Waist to hip ratio was calculated as a marker of abdominal obesity.

Body composition measurements including fat mass (FM), lean mass (LM) and fat percentage were measured and analysed using the Hologic QDR series Discovery A, Bone densitometer [Dual energy X-ray Absorptiometry (DXA)]. Bone mineral density was measured at the femoral neck (FN), lumbar spine (LS) [L1-L4], trochanter, Ward’s triangle and total hip. The in vivo reproducibility of the coefficient of variation ranged between 0.34–0.70% for all measured sites. The DXA machine was calibrated every morning for all the measurements and at the end of each day. The reported spine BMD values were calculated as means of four measured values from L1–L4. Apex System Software version 4.5.3 was used to analyse the DXA scans. Osteoporosis was defined as a T score ≤ − 2·5 and osteopenia as T score between − 1.0 and − 2.5 according to the WHO criteria [25].

### Biochemical blood and bone marker collection and analyses

Plasma samples were collected from centrifuged fasting venous blood samples and stored frozen at -80 °C before analysis. Cytokine assays were prepared using BioLegend® LEGENDplex™ Multi-Analyte Flow Assay kit’s instructions and measured using the Beckman Coulter’s Gallios flow cytometer. Levels of 13 cytokines, namely IL-1β, IFN-α2, IFN-λ, TNF-α, MCP-1, IL-6, IL-8, IL-10, IL-12p70, IL-17A, IL-18, IL-23 and IL-33 were quantified in plasma from the subjects. LEGENDplex™ Data Analysis Software version 8.0 was used to analyze the data. Plasma levels of CRP and ferritin were measured using the electrochemiluminescence immunoassay “ECLIA”. Bone markers C-terminal telopetide (CTX-1) and total procollagen type 1 N-terminal propeptide (P1NP) were analysed by electrochemiluminescence immunoassay using the Roche COBAS® e411 system (Roche Diagnostics, Indianapolis, IN, USA), while vitamin 25(OH)D_3_ was analysed using isotope-dilution liquid chromatography-tandem mass spectrometry (ID-LC-MS-MS) by Canterbury Health, Christchurch, New Zealand.

### Statistical analysis

IBM SPSS version 25 (IBM Company, Armonk, NY, USA) and Minitab were used for the tabular representation of data while R statistical software and Excel were used for the graphical representations. The outcome variables used were spine and hip BMD and T-score. The values of all variables are presented as mean ± standard deviation. One-way analysis of variance (ANOVA) was used to compare the mean values of the CRP quartiles with the subjects’ characteristics. Likewise, ANOVA was used for the comparison of the means of osteoporosis classes (healthy, osteopenia and osteoporosis groups) against levels of cytokines. The mean difference between grouped IL-1β and IL-6 levels and the subjects’ characteristics was determined using the independent t-test. Pearson correlations were used to investigate the relationship between inflammatory markers (continuous) and physiological parameters and bone markers. Partial correlation analyses of the inflammation markers (adjusted for age, height and BMI) were performed against lumbar spine and hip bone mineral content (BMC), BMD, T-score and the bone markers. Correlation results were presented as Pearson correlation coefficient (r) and *p*-value. All *p*-values were reported significant at 0.05 or less.

## Results

The characteristics of the participants who were included in the study are shown in Table 1. Mean (SD) age for participants was 63.2 (4.6) years. The BMI, calculated using the Quetelet’s index, ranged from 14.9 to 38.3 kg/m^2^.

**Table 1:** Characteristics of participants

Parameters (*n* = 86)	Mean ± SD	Min	Max
Age (years)	63.2 ± 4.6	54	81
Weight (kg)	67.3 ± 10.2	43.0	89.2
Height (cm)	161.9 ± 5.2	149.1	1 73.5
BMI (kg/m^2^)	25.7 ± 3.8	14.9	38.3
WC (cm)	79.7 ± 10.9	57.0	110.0
HC (cm)	98.0 ± 7.0	78.0	112.0
WH Ratio	0.8 ± 0.1	0.7	1.0
Spine BMC	53.3 ± 11.6	31.3	82.6
Spine BMD	0.9 ± 0.2	0.5	1.3
Spine T-score	−1.1 ± 1.4	−4.6	2.6
Hip BMC	29.2 ± 5.2	19.0	44.2
Hip BMD	0.8 ± 0.1	0.6	1.1
Hip T-score	−0.8 ± 0.9	−2.5	1.4
Total Fat%	40.4 ± 6.0	14.8	51.1
Total lean mass (kg)	40.1 ± 4.5	30.7	51.2
Ferritin (μg/L)	142.9 ± 100.9	15.00	467.00
25(0H)D_3_ (nmol/L)	78.1 ± 23.5	21.00	160.00

*BMI* Body mass index, *WC* Waist circumference, *HC* Hip circumference, *WH* Waist to hip, *BMC* Bone mineral content, *BMD* Bone mineral density

Subsequently, one-way ANOVA was performed to compare the mean differences according to the quartiles of CRP levels (Table 2). Significant increase in BMI and various body fat percentage was observed as CRP levels increased indicating a positive correlation. On the other hand, the relationship between BMD and T-score with CRP levels was inconsistent and not statistically significant.

**Table 2:** Characteristics according to quartiles of CRP

Parameters	< 0.60 mg/L (*n* = 26)	0.61–1.0 mg/L (*n* = 17)	1.01–2.20 mg/L (*n* = 20)	> 2.21 mg/L (n = 20)	F-value	*P*-value
Age (years)	62.4(4.1)	64.5(6.4)	63.9(4.3)	63.1(3.9)	0.871	0.460
BMI (kg/m^2^)	23.3(3.4)	25.7(3.4)	27.6(2.6)	27.2(4.3)	7.489	**< 0.001**
WC (cm)	73.3(9.7)	80.6(12.0)	84.7(7.2)	83.0(11.3)	5.853	**0.001**
Waist to Hip ratio	0.77(0.07)	0.82(0.09)	0.84(0.06)	0.83(0.09)	4.150	**0.009**
Spine BMD	0.89(0.18)	0.91(0.14)	0.93(0.15)	0.94(0.16)	0.434	0.729
Spine T-score	−1.4(1.6)	−1.2(1.2)	−1.1(1.4)	−0.9(1.3)	0.533	0.661
Hip BMD	0.81(0.12)	0.86(0.12)	0.84(0.12)	0.85(0.08)	0.715	0.546
Hip T-score	−1.27(0.88)	−0.55(1.00)	−0.68(1.06)	− 0.73(0.67)	0.795	0.500
Android fat %	32.3(8.1)	35.0(8.6)	39.9(5.9)	39.3(8.2)	4.795	**0.004**
Gynoid fat %	40.7(7.3)	43.2(4.0)	44.6(3.6)	44.6(4.4)	2.881	**0.041**
Android:Gynoid ratio	0.79(0.13)	0.81(0.17)	0.90(0.12)	0.87(0.14)	2.737	**0.049**
Trunk fat %	32.8(7.1)	36.4(7.3)	40.4(4.2)	40.2(7.5)	6.778	**< 0.001**
Body fat %	36.9(6.9)	40.2(5.7)	42.9(2.9)	42.9(5.6)	6.072	**0.001**
TFM/TLM	0.6(0.2)	0.7(0.2)	0.8(0.1)	0.8(0.2)	7.094	**0.001**
Ferritin (μg/L)	134.0(118.9)	160.4(105.8)	130.5(89.5)	154.1(93.0)	0.398	0.755
25(OH)D_3_ (nmol/L)	83.6(24.8)	80.0(16.6)	75.1(23.6)	73.2(24.8)	0.955	0.418

*BMI* Body mass index, *WC* Waist circumference, *BMD* Bone mineral density, *TFM* Total fat mass, *TLM* Total lean mass, *CRP* C-reactive protein. Variables are reported as mean values with their standard deviation (SD). Number of participants (n). Significant values are in bold text

The relationship between levels of inflammatory markers and osteoporosis status of the participants was also investigated (Table 3). The spine classification was used because of a higher incidence of osteoporosis at the spine than the hip. Mean levels of all the inflammatory markers were higher among the osteoporotic group except in the case of CRP, which was significantly lower in the osteoporotic women, and TNF-α and IL-6 levels which did not show consistent patterns. Meanwhile, there were significant differences for IFN-α2 (*P =* 0.027), IFN-γ (*P =* 0.009), MCP-1 (borderline), IL-12p70 (*P =* 0.049), and IL-33 (*P =* 0.048) across the groups.

**Table 3:** Cytokines/inflammatory markers according to spine osteoporosis status

Markers	Osteoporotic	Osteopenic	Healthy	F-value	*P*-value
	(*n* = 13)	(*n* = 34)	(*n* = 39)		
IL-1β (pg/ml)	7.22(17.59)	2.06(4.88)	1.52(4.21)	2.670	0.075
IFN-α2 (pg/ml)	35.28(48.19)	9.96(18.95)	18.42(26.63)	3.783	**0.027**
IFN-γ (pg/ml)	153.38(222.32)	50.67(58.72)	57.91(73.24)	4.956	**0.009**
TNF-α (pg/ml)	2.01(2.70)	1.83(3.72)	3.19(6.02)	0.795	0.455
MCP-1 (pg/ml)	221.36(100.46)	176.66(43.13)	200.73(55.68)	3.000	**0.055**
IL-6 (pg/ml)	3.22(4.29)	3.14(11.26)	3.09(10.61)	0.001	0.999
IL-8 (pg/ml)	23.85(42.34)	11.19(11.84)	10.66(14.99)	2.220	0.115
IL-10 (pg/ml)	4.62(15.67)	0.57(1.52)	0.75(1.95)	2.261	0.111
IL-12p70 (pg/ml)	6.47(17.03)	1.86(5.74)	0.48(1.36)	3.128	**0.049**
IL-17A (pg/ml)	102.40(227.62)	41.75(79.92)	31.11(45.72)	2.303	0.106
IL-18 (pg/ml)	153.45(72.70)	133.21(69.18)	144.98(51.66)	0.600	0.551
IL-23 (pg/ml)	103.40(173.69)	56.60(91.14)	42.84(55.70)	1.970	0.146
IL-33 (pg/ml)	13.88(37.44)	0.79(3.32)	2.83(11.19)	3.147	**0.048**
CRP (μg/ml)	0.75(1.07)	1.39(1.36)	2.04(2.05)	3.219	**0.044**
Ferritin (μg/L)	149.46(116.16)	124.45(94.57)	156.29(103.72)	0.890	0.415

*IL* Interleukin, *IFN* Interferon, *MCP* Monocyte chemoattractant protein, *CRP* C-reactive protein. Osteoporosis status was according to WHO classification [25]. Variables are reported as mean values with their standard deviation (SD). Number of participants (n). Significant values are in bold text

Based on the hip osteoporosis classification; ANOVA results of MCP-1 were significantly higher in the osteoporotic group compared to the osteopenic and healthy group (*p* = 0.003), however it should be noted that the osteoporotic group was small (*n* = 1).

**Fig. 1:**
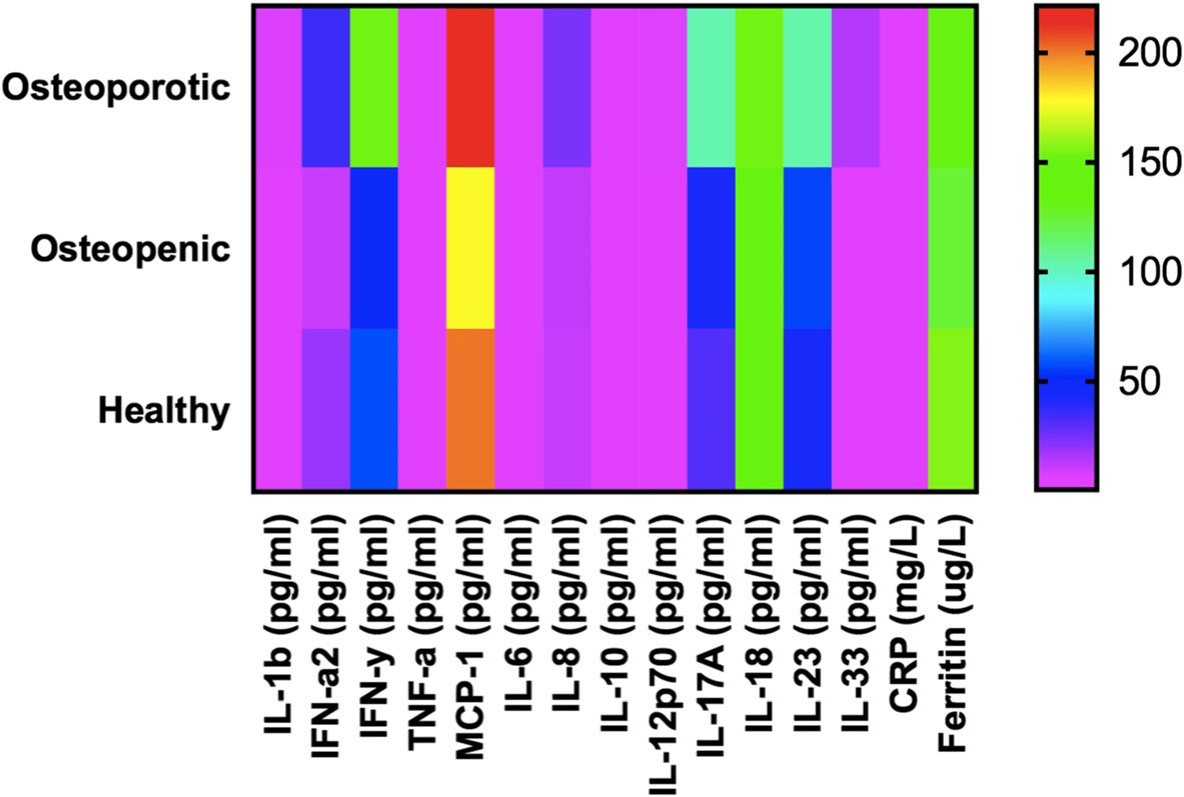
Heat map of inflammatory markers by spine osteoporosis classification

The cytokines and chemokines were generally higher in the osteoporotic group than the healthy/normal or osteopenic groups in this apparently healthy postmenopausal women (Fig. 1).

Investigating the relationship between IL-1β and bone status further, significant differences were found between categories of IL-1β (< 2.39 pg/ml and > 2.39 pg/ml) and spine BMC, BMD, T-score and hip BMD as shown in Table 4. Higher levels of IL-1β were associated with lower BMC, BMD and T-score.

**Table 4:** Independent T-test of characteristics of participants by IL-1β

Variables	IL-1β (< 2.39 pg/ml) [68]	IL-1β (> 2.39 pg/ml) [18]	*P*-value
Age (years)	63.29 ± 4.58	62.89 ± 4.86	0.742
Weight (kg)	67.31 ± 10.28	67.37 ± 10.25	0.981
Height (cm)	161.65 ± 5.19	162.57 ± 5.20	0.506
BMI (kg/m^2^)	25.77 ± 3.87	25.48 ± 3.62	0.770
WC (cm)	79.79 ± 10.92	79.37 ± 11.10	0.885
Waist-Hip ratio	0.81 ± 0.08	0.81 ± 0.08	0.990
Spine BMD	0.94 ± 0.16	0.85 ± 0.14	**0.028**
Spine T-score	−0.94 ± 1.43	−1.77 ± 1.26	**0.021**
Femoral Neck BMD	0.71 ± 0.10	0.66 ± 0.08	**0.019**
Hip BMD	0.85 ± 0.12	0.79 ± 0.09	**0.020**
Hip T-score	−0.73 ± 0.95	−1.16 ± 0.86	0.071
Lean mass (kg)	40.07 ± 4.43	40.37 ± 4.78	0.798
Total Fat%	40.34 ± 6.22	40.40 ± 5.36	0.967
Ferritin (μg/L)	145.03 ± 107.45	134.28 ± 81.40	0.647
25(OH)D_3_ (nmol/L)	78.32 ± 29.94	77.00 ± 18.62	0.806

*IL* Interleukin, *BMI* Body mass index, *WC* Waist circumference, *BMC* Bone mineral content, *BMD* Bone mineral density, *CRP* C-reactive protein. Variables are reported as mean values with their standard deviation (SD). Number of participants [n]. Significant values are in bold text

Table 5 shows the relationship between IL-6 and participants’ characteristics indicating significant differences between IL-6 and weight, waist circumference as well as the hip BMC and BMD. There were higher levels of IL-6 found in individuals with lower BMC, BMDs and T-scores.

**Table 5:** Independent T-test of participants’ characteristics by IL-6

Variables	IL-6 (< 1.62 pg/ml) [53]	IL-6 (> 1.62 pg/ml) [33]	*P*-value
Age (years)	63.34 ± 4.86	63.00 ± 4.24	0.734
Weight (kg)	65.66 ± 10.05	70.00 ± 10.03	**0.054**
Height (cm)	161.66 ± 5.01	162.15 ± 5.49	0.672
BMI (kg/m^2^)	25.09 ± 3.47	26.69 ± 4.16	0.057
WC (cm)	77.59 ± 9.77	83.11 ± 11.88	**0.021**
Waist-Hip ratio	0.80 ± 0.07	0.84 ± 0.09	**0.020**
Spine BMD	0.94 ± 0.15	0.90 ± 0.17	0.343
Spine T-score	−0.99 ± 1.37	−1.32 ± 1.52	0.291
Femoral Neck BMD	0.71 ± 0.10	0.67 ± 0.09	**0.035**
Hip BMD	0.86 ± 0.12	0.81 ± 0.10	**0.033**
Hip T-score	−0.64 ± 0.98	−1.10 ± 0.82	**0.023**
Lean mass (kg)	39.55 ± 4.59	41.07 ± 4.18	0.117
Total Fat%	39.55 ± 6.37	41.65 ± 5.25	0.100
Ferritin (μg/L)	142.75 ± 107.04	143.12 ± 91.74	0.987
25(OH)D_3_ (nmol/L)	80.17 ± 24.14	74.85 ± 22.46	0.303

*IL* Interleukin, *BMI* Body mass index, *WC* Waist circumference, *BMC* Bone mineral content, *BMD* Bone mineral density, *CRP* C-reactive protein. Variables are reported as mean values with their standard deviation (SD). Number of participants [n]. Significant values are in bold text

Table 6 shows the correlation coefficients of the inflammatory markers against bone health indicators. Significant negative correlations were found between IL-12p70 and bone health parameters as well as between CRP and the bone turnover markers P1NP and CTX-1. In Table 6, vitamin 25(OH)D_3_ was in general significantly positively correlated with inflammatory markers. An evidence of vitamin D as a stimulant of the innate immune system.

**Table 6:** Correlations of inflammatory markers with bone health indicators across groups

	Spine BMD	Spine T-score	Hip BMD	Hip T-score	P1NP (ug/L)	CTX-1 (ug/L)	PTH (pmol/L)	25(OH)D_3_ (nmol/L)
IL-1β (pg/ml)	− 0.211*	−0.216*	− 0.181	− 0.154	0.010	− 0.049	− 0.027	0.092
IFN-α2 (pg/ml)	− 0.065	− 0.076	− 0.130	− 0.138	− 0.154	− 0.226*	0.062	0.051
IFN-γ (pg/ml)	−0.106	− 0.115	− 0.181	− 0.175	− 0.125	− 0.189	− 0.005	0.218*
TNF-α (pg/ml)	0.153	0.151	0.075	0.068	− 0.020	− 0.003	0.068	−0.067
MCP-1 (pg/ml)	0.049	0.035	−0.024	− 0.023	− 0.117	−0.124	0.013	0.287**
IL-6 (pg/ml)	−0.018	− 0.036	− 0.089	− 0.094	− 0.207	− 0.145	− 0.033	0.127
IL-8 (pg/ml)	−0.120	− 0.128	− 0.164	−0.155	0.039	−0.049	− 0.105	0.209
IL-10 (pg/ml)	−0.172	− 0.177	− 0.157	− 0.164	− 0.051	− 0.142	− 0.104	0.185
IL-12p70 (pg/ml)	−0.220*	− 0.227*	− 0.241*	− 0.250*	− 0.103	−0.192	− 0.148	0.195
IL-17A (pg/ml)	−0.182	− 0.189	− 0.123	− 0.092	− 0.009	−0.091	− 0.180	0.245*
IL-18 (pg/ml)	0.023	0.029	−0.069	− 0.075	− 0.160	−0.143	− 0.002	0.097
IL-23 (pg/ml)	−0.176	− 0.185	− 0.131	−0.090	− 0.026	−0.046	− 0.073	0.246*
IL-33 (pg/ml)	−0.098	−0.102	− 0.161	−0.167	− 0.026	−0.087	− 0.074	0.234*
CRP (μg/ml)	0.190	0.196	0.148	0.164	−0.253*	−0.223*	0.132	−0.132
Ferritin (μg/L)	0.190	0.228*	0.178	0.197	0.080	0.064	−0.156	0.024

* p < .05, **p < .01. *IL* Interleukin, *IFN* Interferon, *TNF* Tumour necrosis factor, *MCP* Monocyte chemoattractant protein, *CRP* C-reactive protein, *BMD* Bone mineral density. Significant values are in bold text

Partial correlations adjusted for the effect of age and BMI on the relationship between inflammatory markers and bone health were explored in Table 7. IL-6 was significantly negatively correlated with hip BMD, hip T-score and P1NP. Vitamin 25(OH)D_3_ was positively correlated with MCP-1, IL-6, IL-17A and IL-23.,

**Table 7:** Partial correlations of inflammatory markers with bone health indicators adjusting for age and BMI

	Spine BMD	Spine T-score	Hip BMD	Hip T-score	P1NP (ug/L)	CTX-1 (ug/L)	PTH (pmol/L)	25(OH)D_3_ (nmol/L)
IL-1β (pg/ml)	−0.188	− 0.197	− 0.217	− 0.188	0.010	−0.064	− 0.039	0.092
IFN-α2 (pg/ml)	−0.003	− 0.017	− 0.075	− 0.087	− 0.185	− 0.222*	0.065	0.054
IFN-γ (pg/ml)	0.020	0.008	−0.103	− 0.101	− 0.145	−0.221*	0.028	0.174
TNF-α (pg/ml)	0.183	0.180	0.102	0.092	−0.009	− 0.012	0.064	− 0.079
MCP-1 (pg/ml)	0.084	0.067	0.024	0.027	−0.086	− 0.154	0.037	0.277**
IL-6 (pg/ml)	−0.105	−0.131	− 0.256*	− 0.266*	− 0.222*	− 0.166	− 0.107	0.228*
IL-8 (pg/ml)	0.015	0.005	−0.093	−0.087	0.004	−0.081	− 0.093	0.190
IL-10 (pg/ml)	−0.100	−0.108	− 0.160	− 0.174	− 0.069	− 0.163	− 0.115	0.190
IL-12p70 (pg/ml)	−0.094	− 0.102	− 0.185	− 0.202	− 0.156	− 0.228*	− 0.128	0.158
IL-17A (pg/ml)	−0.057	− 0.067	− 0.049	− 0.011	− 0.062	−0.123	− 0.167	0.240*
IL-18 (pg/ml)	0.109	0.117	− 0.016	− 0.025	− 0.163	− 0.169	− 0.007	0.124
IL-23 (pg/ml)	−0.051	−0.062	− 0.039	0.010	− 0.079	−0.076	− 0.050	0.232*
IL-33 (pg/ml)	0.016	0.011	−0.096	−0.108	− 0.039	−0.102	− 0.053	0.205
CRP (μg/ml)	0.004	0.009	−0.077	−0.053	− 0.257*	−0.256*	0.072	−0.030
Ferritin (μg/L)	0.112	0.157	0.136	0.160	0.093	0.058	−0.191	0.049

* p < .05, **p < .01. *IL* Interleukin, *IFN* Interferon, *TNF* Tumour necrosis factor, *MCP* Monocyte chemoattractant protein, *CRP* C-reactive protein, *BMD* Bone mineral density. Significant values are in bold text

Furthermore, as shown in Table 8, partial correlations adjusted for age and height (to reflect BMC which varies with height) indicated stronger relationships between the 15 markers of inflammation and bone, especially negative correlations between IL-1β and IL-12p70 and spine BMC/spine BMD/spine T-score. In addition, Vitamin 25(OH)D_3_ was positively correlated with IFN-γ, MCP-1, IL-8, IL-10, IL-12p70, IL-17A, IL-23 and IL-33.

**Table 8:** Partial correlations of inflammatory markers with bone health indicators adjusting for age and height

	Spine BMC	Spine BMD	Spine T-score	Hip BMC	Hip BMD	Hip T-score	P1NP (ug/L)	CTX-1 (ug/L)	PTH (pmol/L)	25(OH)D_3_ (nmol/L)
IL-1β (pg/ml)	−0.224*	− 0.221*	− 0.229*	− 0.209	− 0.264*	− 0.238*	0.000	− 0.079	− 0.054	0.106
IFN-α2 (pg/ml)	−0.015	− 0.031	− 0.044	− 0.072	− 0.109	− 0.121	− 0.198	− 0.238*	0.057	0.058
IFN-γ (pg/ml)	− 0.111	− 0.117	− 0.129	− 0.203	− 0.260*	−0.258*	− 0.158	− 0.245*	− 0.020	0.220*
TNF-α (pg/ml)	0.182	0.157	0.154	0.073	0.050	0.039	−0.047	− 0.050	0.073	− 0.155
MCP-1 (pg/ml)	0.004	0.022	0.005	−0.015	− 0.072	− 0.070	− 0.125	− 0.200	0.029	0.267*
IL-6 (pg/ml)	−0.120	0.000	−0.022	− 0.075	− 0.123	− 0.133	− 0.222*	− 0.156	− 0.067	0.165
IL-8 (pg/ml)	−0.089	− 0.118	− 0.128	−0.194	− 0.258*	− 0.253*	− 0.024	− 0.121	− 0.131	0.223*
IL-10 (pg/ml)	− 0.173	− 0.166	− 0.174	− 0.229*	− 0.232*	− 0.244*	− 0.067	− 0.169	− 0.141	0.225*
IL-12p70 (pg/ml)	−0.257*	− 0.226*	− 0.234*	− 0.338**	− 0.331**	− 0.344**	− 0.152	− 0.236*	− 0.173	0.222*
IL-17A (pg/ml)	−0.197	− 0.188	− 0.198	− 0.248*	− 0.220	−0.186	− 0.082	−0.156	− 0.205	0.278*
IL-18 (pg/ml)	0.087	0.040	0.046	−0.019	−0.116	− 0.125	− 0.205	− 0.216	−0.018	0.117
IL-23 (pg/ml)	−0.193	−0.178	− 0.190	−0.248*	− 0.217	−0.172	− 0.117	− 0.125	− 0.087	0.257*
IL-33 (pg/ml)	−0.094	− 0.096	− 0.101	−0.187	− 0.213	−0.223*	− 0.039	−0.112	− 0.092	0.253*
CRP (μg/ml)	0.142	0.171	0.177	0.061	0.137	0.157	−0.233*	−0.216	0.127	−0.105
Ferritin (μg/L)	0.182	0.182	0.223*	0.261*	0.197	0.218	0.066	0.041	−0.145	−0.027

* *p* < .05, **p < .01. *IL* Interleukin, *IFN* Interferon, *TNF* Tumour necrosis factor, *MCP* Monocyte chemoattractant protein, *CRP* C-reactive protein, *BMC* Bone mineral content, *BMD* Bone mineral density. Significant values are in bold text

**Fig. 2:**
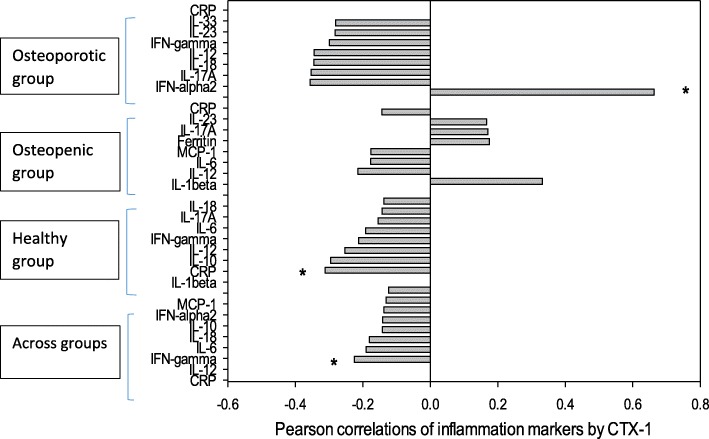
Pearson correlations of inflammatory markers by CTX-1

Figure 2 shows the Pearson correlations of inflammatory markers against CTX-1 when separated into the osteoporotic, osteopenic, healthy and across all groups. There is a significant positive correlation between IFN-α2 and CTX-1 amongst the osteoporotic group, and a negative correlation between CRP and CTX-1 in the healthy group.

### The relationship between inflammatory markers and fat percentage across groups

**Fig. 3:**
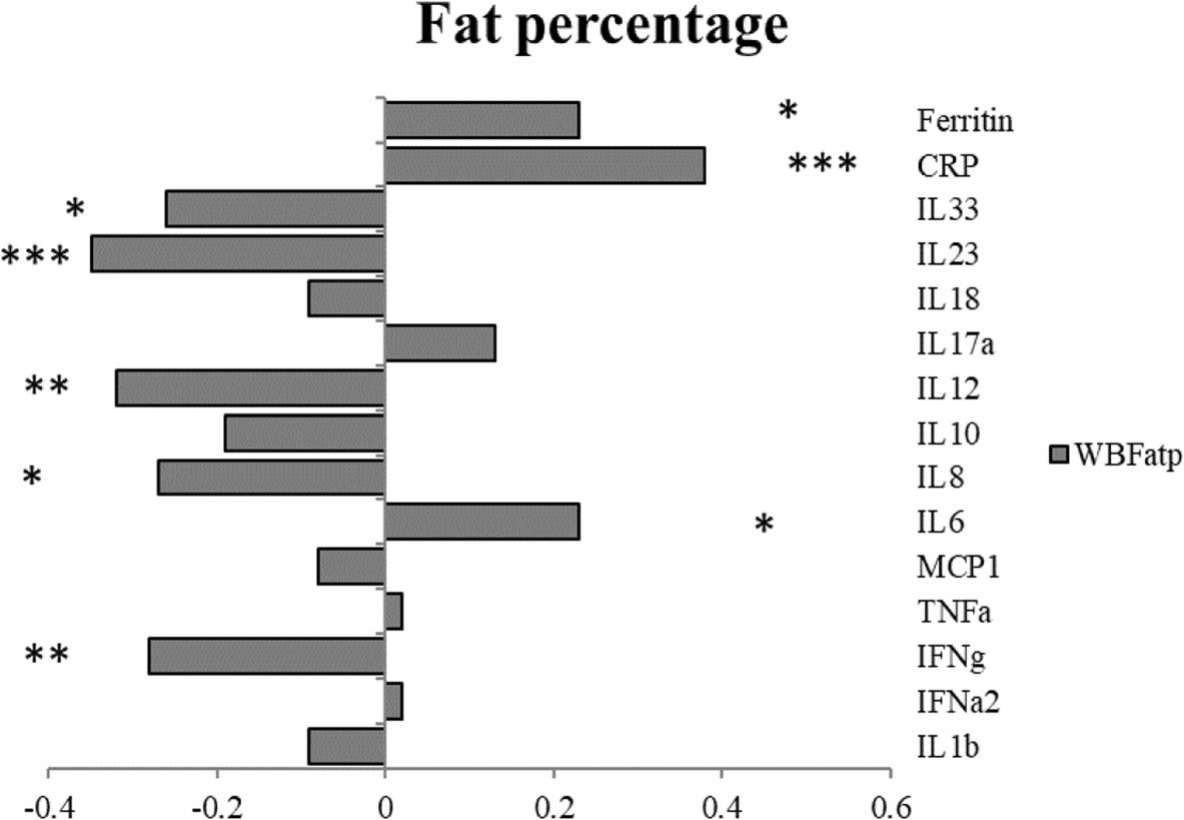
Correlations of inflammation markers by fat percentage. * *p* < .05, **p < .01, *** *p* < .001

The bar graphs in Fig. 3 show the correlations between the 15 inflammatory markers and fat percentage. Levels of CRP were the most strongly positively correlated with whole body fat percentage (WBFatp), as oppose to IL-23 which was strongly negatively correlated.

From Fig. 3, we observed significant positive correlations between ferritin, CRP and IL-6 and the fat percentage. All other cytokines were negatively correlated with the fat percentage, with statistically significant correlations for IL-33, IL-23, IL-12, IL-8 and IFN-γ.

### Ferritin as a marker of inflammation

Analysis of anti-inflammatory cytokines’ production against ferritin levels indicated significant differences between IL-10 (*p*-value = 0.012), IL-33 (p-value = 0.017) and the levels of ferritin.. High levels of ferritin were significantly associated with low levels of IL-10 and IL-33, indicating a possible anti-inflammatory effect of IL-10 and IL-33 on the ferritin status of individuals.

In summary, these results provide important insights into a cross-sectional overview of inflammatory markers and bone health during midlife and senescence. Thereby, providing platforms for further hypotheses and research in this area.

## Discussion

In general terms, the cytokines measured in this study can be described as

1. Inflammatory (osteoclastogenic) cytokines include IL-1β, IL-6, IL-8, IL-17, MCP-1, TNFα, IFN-α2 and IFN-γ). They are generally known for their degenerating and catabolic effects on tissue metabolism and homeostasis as well as the intracellular actions and signalling pathway to osteoclastic differentiation [26]. More so, in postmenopausal women, when coupled with the effect of oestrogen deficiency. Elevated levels of inflammatory cytokines have been linked with lower bone mineral density [12], as part of “inflammaging” [27].
2. Anti-Inflammatory (anti-osteoclastogenic) cytokines include IL-10, IL-18, IL-33. Most often anti-inflammatory cytokines exert opposite effects to those of inflammatory cytokines on bone.
3. Dual-role cytokines include IL-12 and IL-23. In certain circumstances, these cytokines provide a balancing act, with a dual-role in the regulation of the immune system.

It must be appreciated that although individual cytokines may be described as pro- or anti-inflammatory, they interact closely with one another as part of a dynamic network which creates a balance of both inhibitory and stimulatory immune effects [26].

Some studies, although limited and contradictory, have investigated the impact of immune cytokines, especially IL-6, on bone loss in postmenopausal women [28–32]. However, to our knowledge no study has investigated the relationship of all the 15 immune markers with bone health in postmenopausal women.

### CRP

CRP is a sensitive marker of systemic inflammation. The production of CRP in the liver upregulates other inflammatory cytokines (including IL-1, IL-6 and TNFα) and has been shown to be positively correlated with bone resorption and hip and spinal bone loss in healthy pre- and post-menopausal women [7–9].

In our study CRP was significantly associated with measures of BMI and various measures of body fat. High BMI, body fat and fat percentage was associated with high CRP, supporting previous studies linking obesity with increased systemic inflammation [33]. These results are similar to those of the Dunedin study that reported a positive correlation between CRP and BMI [34]. Similar results to our findings was also reported by Berglundh et al. 2015, their results indicated that women with lower CRP quartiles had lower BMD values [8].

However, in contrast to the literature, CRP was lower in the osteoporotic women than those with osteopenia or healthy bone. This may reflect a lower body weight in women with osteoporosis or may be an effect of the small number of women with osteoporosis in this study. In addition, CRP was negatively correlated with both P1NP (a bone formation marker) and CTX-1 (a bone resorption marker), indicating a lower overall bone turnover with high levels of CRP. However, the findings of this study is in accordance to that of Huang and Schooling which found no association between higher hsCRP and lower BMD [35].

Of interest also was the observation that higher levels of CRP were associated with low vitamin 25(OH)D_3_ levels. Although this relationship did not reach statistical significance it is potentially worth investigating further in future studies.

### Immune cytokines and bone health

With participants grouped to healthy, osteopenic and osteoporotic groups based on spine BMD measurements, (Table 3) the means of all immune markers (with the exception of CRP, discussed above) were higher in the osteoporotic group, and ANOVA demonstrated significant differences for IFN-α2, INF-γ, IL-12P70, IL-33, and trending increased in IL-1β, MCP-1, and IL-23. This increase in both pro- and anti-inflammatory markers may indicate a more active upregulated immune status in those with poor bone health.

Unexpectedly, we observed higher levels of IL-10 (an osteoprotective cytokine) in the osteoporotic group and correlations with bone health indicators were negative. In addition, although IL-33 has been reported as an anti-osteoclastogenic cytokine [6, 36], we found levels of IL-33 to be significantly higher in the osteoporotic group than the normal or osteopenia groups, and correlations with bone health parameters were negative although they did not reach statistical significance. The negative correlations between IL-33 and bone resorption marker CTX as reported by Ginaldi et al. (2019) is however similar to the result of this study [36]. However, further studies with larger numbers of women are needed in this area.

In agreement with the literature [29, 37, 38], independent T tests showed a significant negative relationship between IL-1β and bone parameters – high pro-inflammatory IL-1β was associated with lower BMC, BMD and T-scores (Table 4). For proinflammatory IL-6, there was a positive association with BMI, WC and waist-hip ratio, and a negative association with femoral neck BMD, hip BMD and hip T-score.

### Correlations

Analysis of correlations between bone parameters and immune markers (Table 6) showed a significant negative correlation between IL-12p70 and spine and hip bone measurements. Adjustment for age and height retained this relationship, as well as showing a significant negative relationship between IL-1β and spine and hip bone measurements. The other associations with hip measurements are not discussed further due to low numbers of women with poor hip bone health. Additional data are needed to confirm these associations.

Of note from these correlations are the associations of immune markers with bone biomarkers: IFN-α2, IFN-γ, and IL-12p70 had a negative association with CTX-I, so high levels of potentially inflammatory cytokines were associated with low CTX-1 and hence low bone resorption activity.

Similarly, IL-6 showed a negative correlation with P1NP, so high levels of pro-inflammatory IL-6 were associated with low levels of bone formation.

Evidence for the anti-inflammatory effects of vitamin 25(OH)D_3_ were observed in its correlations with the inflammatory markers. Vitamin 25(OH)D_3_ was positively correlated with all the inflammatory cytokines except TNF-α and CRP.

### Inflammatory markers and body fat percentage

Body fat and obesity have been reported to be positively associated with low-grade inflammation [33, 39]. This was further illustrated in our study, where BMI and various body fat measurements (including fat mass, fat percentage, abdominal fat, waist circumference, waist to hip ratio, android fat, gynoid fat, android to gynoid fat ratio, trunk fat percentage, and body fat percentage, and fat mass to lean mass ratio) all had significant positive correlations with CRP and IL-6 (both markers of inflammation).

In addition, we found significant negative correlations between several of the potentially anti-inflammatory markers (IL-23, IL33, and IL-12) and body fat percentage in all groups of women. The proinflammatory markers IL-8 and IFN-γ also had negative correlations with body fat percentage, which may reflect the complex cytokine interactions regulating/balancing the immune system. In general, high body fat percentage indicating obesity appears to be associated with increased inflammation and lower levels of anti-inflammatory cytokines.

### Ferritin and anti-inflammatory cytokines

Infection, injury or trauma influences iron status. However, iron in the body is stored as ferritin in normal healthy individuals. Serum ferritin concentration is well-known as an important, convenient and accurate indicator of total body iron stores in humans. Hepcidin hormone is a major regulator of systemic iron homeostasis in the liver and it is induced during inflammation. Thus, ferritin is also known as an acute phase reactant, a marker of inflammation.

Hypoferremia and a high serum ferritin concentration during acute phase response has been linked to the actions of pro-inflammatory cytokines both in vivo and in vitro [40]. High levels of ferritin have been suggested to act as a potent oxidant causing oxidative stress which is associated with increased risk of various diseases [41]. Thus, iron levels must be regulated and controlled in order to provide an essential nutrient that is capable of oxygen delivery and metabolism with regulated redox reactions, but without causing cellular damage or apoptosis by guarding against excessive toxicities [42, 43].

In the liver, during infection with extracellular bacteria, hepcidin targets ferroportin (FP1) [a transmembrane protein that transports iron from the inside of a cell to the outside] and results in its degradation which causes a reduction in hepcidin levels produced by macrophages as a result of inflammatory cytokines; giving rise to hypoferremia (reduced iron availability for extracellular bacteria) and high levels of ferritin [44].

In an experimental study by Feelder et al., following an administration of inflammatory cytokines (TNF-α, IL-1 and IL-6), an increase in ferritin synthesis and a decrease in serum iron were observed [40]. Furthermore, Nairz et al. reported that in response to the actions of pro-inflammatory cytokines such as IL-6 and IFN-γ, systemic hypoferremia and increased ferritin were observed as a result of extracellular actions [44].

Furthermore, ferritin has been postulated as a disease marker and mediator [45]. However, contrary to our findings, ferritin has been suggested to be responsible for immune suppression via increased production of IL-10 in the presence of chemokines [45].

The results from our cross-sectional study showed that anti-inflammatory cytokines IL-10 and IL-33 are significantly related to lower plasma ferritin status in a cohort of post-menopausal women. This suggests a possible role of anti-inflammatory, anti-osteoclastogenic cytokines in iron metabolism and the regulation of iron stores. Further research work is needed to confirm the role of anti-inflammatory cytokines in iron homeostasis.

Overall, our findings showed that in a group of apparently healthy postmenopausal women, cytokine levels of IFNα2, IFN-γ, IL-12p70, IL-33 and MCP-1 were significantly higher in groups with low bone mass (osteoporosis) than those with higher bone mass (osteopenia and healthy). We also found that when converted into categorical data, there were significant differences in levels of IL-1β according to the spine and hip classifications. Similarly, we observed significant differences in levels of IL-6 by the hip categorical classification. Partial correlation coefficients with adjustment for age and height showed negative correlations between IL-1β, IFN-α2, IFN-γ, IL-6, IL-8, IL-10, IL-12p70, IL-23, IL-33 and hip and spine BMC, BMDs, and T-scores. Furthermore, we found that high levels of ferritin were significantly associated with low IL-10 and IL-33 levels in these postmenopausal women.

Limitations of this study include the cross-sectional nature preventing any cause and effect relationship and the small number of osteoporotic postmenopausal women compared to the healthy and osteopenic groups.

## Conclusion

In summary, although specific individual cytokines were correlated with bone health (most clearly demonstrated by the observation that high levels of IL-β were associated with low spine and hip bone measurements), many cytokines (both pro- and anti-inflammatory) were shown to be elevated in osteoporotic women, perhaps indicating upregulation of the overall immune system in women with poor bone health.

Some inflammatory markers were also shown to have an impact on bone turnover: CRP was negatively correlated with both P1NP (a bone formation marker) and CTX-1 (a bone resorption marker), indicating lower overall bone turnover with high levels of CRP. High levels of potentially inflammatory cytokines (IFN-α2, IFN-γ, and IL-12p70) were associated with low CTX-1 and hence low bone resorption activity, and high levels of pro-inflammatory IL-6 were associated with low levels of the bone formation marker P1NP. Of note also, high fat percentage indicating obesity was associated with increased inflammation and lower levels of anti-inflammatory cytokines.

This study strengthens the knowledge of the role of immune factors in promoting bone degradation in older women. Despite these promising results, to develop a full picture of the impact of cytokines and chemokines on bone health, additional longitudinal intervention studies are recommended to confirm and expand the relationships described in this study.

## Data Availability

The datasets used and/or analysed during the current study are available from the corresponding author on reasonable request.

## Abbreviations

BMC: Bone mineral content
BMD: Bone mineral density
CRP: C-reactive protein
IFN: Interferon
IL: Interleukin
MCP: Monocyte chemoattractant protein
OC: Osteoclast
TNF: Tumour necrosis factor
